# ^18^F-FDG PET/CT在419例肺外科患者中的应用经验

**DOI:** 10.3779/j.issn.1009-3419.2012.01.05

**Published:** 2012-01-20

**Authors:** 菲 王, 少华 马, 潞艳 申, 囡 李, 志 杨, 克能 陈

**Affiliations:** 1 100142 北京，北京大学肿瘤医院暨恶性肿瘤发病机制及转化研究教育部重点实验室胸外一科 Department of Thoracic Surgery Ⅰ, Key Laboratory of Carcinogenesis and Translational Reaserch (Ministry of Education), Peking University Cancer Hospital, Beijing 100142, China; 2 100142 北京，北京大学肿瘤医院暨恶性肿瘤发病机制及转化研究教育部重点实验室核医学科 Department of Nuclear Medicine, Key Laboratory of Carcinogenesis and Translational Reaserch (Ministry of Education), Peking University Cancer Hospital, Beijing 100142, China

**Keywords:** 肺肿瘤, 肿瘤样病变, PET/CT, Lung neoplasms, Tumor-like disease, PET/CT

## Abstract

**背景与目的:**

我国是肺癌的高发国家，PET/CT在我国的肺外科临床应用价值仍处在探索阶段。本研究总结病理证实的419例肺部肿瘤或肿瘤样病变的患者资料，探讨PET/CT在这一领域的应用价值。

**方法:**

2007年12月-2011年8月北京大学肿瘤医院胸外一科单个医疗组对594例肺部肿瘤或肿瘤样病变的患者在诊治过程的不同阶段进行了PET/CT检查，以获得病理的419例患者为研究对象，结合临床、病理及随访，分析PET/CT在良恶性定性，肺癌TNM分期，疗效评价及疗后随访中的应用价值。

**结果:**

全组419例患者中病理证实为良性者63例，恶性者356例，其中原发性肺癌338例，肺转移瘤18例。PET/CT对恶性肿瘤定性诊断（SUVmax＞2.5）的敏感性为85.0%，特异性为52.4%，准确性79.2%，阳性预测值89.2%，阴性预测值42.9%。338例患者中治疗前行PET/CT者275例，共发现远处转移46例（46/275, 16.7%），较传统检查（38/275, 13.8%）多发现8例。对临床怀疑术后复发者（89例）行PET/CT，发现复发43例（43/89, 48.3%），较传统检查（37/89, 41.6%）多发现6例。对手术的168例患者作了T及N分期相关性的研究，发现SUVmax与肿瘤直径正相关（*P*＜0.05）。清扫淋巴结共计610组，PET/CT诊断肺癌淋巴结转移的敏感性为36.3%，特异性为93.9%，准确性为84.3%，阳性预测值为54.4%，阴性预测值为88.0%。全组有10例患者化疗前后均作了PET/CT，SUVmax随肿瘤降期而下降，下降平均百分比为37.5%（*P*＜0.05）。

**结论:**

PET/CT是现阶段除组织学外另一种可选的判断肺部良恶性病变的方法。PET/CT在肺癌M分期中的作用优于传统检查；也可作为术后复查的常规手段之一。PET/CT对淋巴结转移的诊断特异性较好，但敏感性不高。PET/CT在肺癌化疗评效中有积极意义。

## Abstract (en)

**Background and objective:**

The application and the value of PET/CT in lung cancer are on the way to development. The aim of this study is to summarize the data from 419 patients with pulmonary tumor or tumor-like disease and to explore the appliance of PET/CT.

**Methods:**

From Dec 2007 to Aug 2011, 594 patients with pulmonary tumor or tumor-like disease underwent PET/CT examination during the different course of treatment, which were treated by single surgery team from Peking University Cancer Hospital. Of these patients, 419 cases diagnosed pathologically were included into study. The clinicapathological and follow-up data were collected to analyze the value of PET/CT in diagnosis, TNM staging, therapy response evaluation and posttherapy monitoring.

**Results:**

Four hundred and nineteen cases comprised of 63 benign and 356 malignant, of which, 338 were primary lung cancer, and 18 were metastases. The SUVmax cutoff was defined as 2.5 to differentiate the benign and malignant disease. PET/CT obtained the role of diagnosis with sensitivity as 85.0%, specificity as 52.4%, accuracy rate as 79.2%, positive predictive value as 89.2% and negative predictive value as 42.9%, respectively. Of 338 primary cases, 275 underwent PET/CT at initial diagnosis, in which, 46 (16.7%) distant metastasis were found, including 8 additional metastasis not found by conventional utilities. Six of 43 recurrences were found by PET/CT following conventional examination. In T staging, SUVmax is positively correlated with diameter of tumors (*P* < 0.05). In N staging, 610 stations of lymph nodes were resected from 168 cases, with 37 stations predicted as positive by PET/CT and 102 stations pathologically proven positive, therefore, calculating the sensitivity as 36.3%, specificity as 93.9%, accuracy rate as 84.3%, positive predictive value as 54.4% and negative predictive value as 88.0%, respectively. Ten patients underwent PET/CT scan for chemotherapy response evaluation, with SUVmax changing following T downstaging.

**Conclusion:**

PET/CT is one optional method for diagnosis of pulmonary tumors. In TNM staging, PET/CT showed the superiority than conventional utilities in M staging, but possesses the high specificity but inferior sensitivity in N staging. Therefore, PET/CT should be used as routine examination for postoperation follow-up. Furthermore, PET/CT performed the outstanding role in chemotherapy response evaluation.

肺癌的发病率和死亡率在我国均居恶性肿瘤第1位，其诊断、分期及疗效评估仍是胸部肿瘤外科医生面临的主要问题之一。传统方法包括胸片、胸部CT、头颅MRI、气管镜、超声、骨扫描等，这些检查方法多基于单一解剖结构学或功能学，准确性有限。PET/CT是首个将功能与结构相结合的影像检查手段，为肺癌的诊治提供了重要的帮助。但PET/CT在国内的应用尚处在初级阶段，颇有争议，需要探讨。本文总结北京大学肿瘤医院胸外一科单个医疗组收治的有病理证实的419例肺部肿瘤或肿瘤样病变患者，将PET/CT检查结果与病理、临床和随访进行对照研究，旨在探讨PET/CT在肺部肿瘤或肿瘤样病变定性诊断及肺癌分期和疗效评估及随访中的应用价值。

## 1 资料与方法

### 1.1 一般资料

2007年12月-2011年8月北京大学肿瘤医院胸外一科单个医疗组共对594例肺部肿瘤或肿瘤样病变患者实施了PET/CT检查。175例无病理诊断，另419例有病理诊断（支气管镜/CT引导穿刺/淋巴结活检/手术），该419例为本文的研究对象。全组419例中男性250例，女性169例，年龄12岁-81岁，中位年龄59岁。良性病变63例，包括炎性肉芽肿21例，错构瘤11例，非特异性炎症14例，炎性假瘤3例，硬化性血管瘤4例，其它良性病变病理类型10例，病理获得方式均为手术切除。419例中恶性肿瘤356例，包括肺恶性转移瘤18例均行手术切除（包括软组织肉瘤/骨肉瘤肺转移7例，结直肠癌肺转移9例，乳腺癌肺转移2例），原发肺癌者338例，其中双原发癌10例（食管癌并肺癌2例，胃癌并肺癌2例，肺癌术后第二原发肺癌6例）。338例肺癌中231例获得病理方式为手术切除（局部切除术20例、肺叶切除术193例、全肺切除术11例、袖状肺叶切除术7例），其余107例获取病理的方式为支气管镜42例，CT引导穿刺32例，淋巴结活检24例，其它方法9例。338例中腺癌194例，鳞癌78例，小细胞癌23例，其它病理类型43例（表 1）。

**1:** 419例患者临床资料 Clinical characteristics of 419 patients

Variable	*n*	%
Gender		
Male	250	59.7
Female	169	40.3
Media age (yr)	59	
Pathology		
Benign	63	
Granulomatous inflammation	21	33.3
Hamartoma	11	17.5
Nonspecific inflammation	14	22.2
Inflammatory pseudotumor	3	4.8
Sclerosing hemangioma	4	6.3
Others	10	15.9
Malignancy	356	
Squamous carcinoma	78	21.9
Adenocarcinoma	194	54.5
Small cell carcinoma	23	6.5
Metastatic tumor	18	5.1
Others	43	12.0

### 1.2 PET/CT成像方法

2007年12月-2009年12月患者^18^F-FDG PET/CT扫描采用西门子公司PET/CT显像仪（Biograph HR 16, Siemens, German），其中PET部分采用LSO晶体和微电子设备，CT部分为16排高分辨螺旋CT。2009年12月-2011年8月^18^F-FDG PET/CT扫描采用飞利浦公司PET/CT显像仪（Gemini TF 16, Philips, Netherland），其中PET/CT部分采用LYSO晶体和微电子设备，CT部分为16排高分辨螺旋CT。所有患者检查前空腹6 h以上。测定患者空腹血糖水平＜10 mmol/L。按患者体重（3.0 MBq/kg-3.7 MBq/kg）静脉注射^18^F-FDG后，患者平静休息，60 min左右行颅脑及躯干部PET/CT图像采集。发射采集大多数从床尾开始，每床位扫描1.5 min-2 min。由2位以上经验丰富的核医学专家阅片并行图像分析。观察指标选取肿瘤最大标准摄取值SUVmax（maximum standardized uptake value, SUVmax）。

### 1.3 PET/CT检查资料

PET/CT用于恶性肿瘤术后复查者89例，419例中可用于定性分析共计356例，其中338例肺癌中治疗前行PET/CT者275例（手术治疗168例，其它107例）用于M分期的分析，其中168例手术切除者用作T分期及N分期的分析。89例用于肺癌术后随访，包括术前术后均行PET/CT者26例。全组用于化疗前后评效者10例。

### 1.4 随访

北京大学肿瘤医院胸外一科单个医疗组的肺癌术后随访率为95%，术后2年内每3个月、2年-5年内每6个月、5年后每年各随访1次。随访方法主要以门诊全面复查为主，检查内容包括胸片、胸部CT、颈部及腹部超声、头颅MRI、全身骨扫描及化验检查。此次总结时门诊未随访到的患者进行电话随访、书信随访及家访。全组末次随访时间为2011年11月15日。

### 1.5 统计分析

统计软件采用SPSS 13.0软件包。肿瘤SUVmax与肿瘤大小相关分析采用*Spearman*相关分析。化疗前后SUVmax差异和PR与SD患者SUVmax下降百分比之间的差异采用配对样本*t*检验，*P*＜0.05为差异有统计学意义。

## 2 结果

### 2.1 PET/CT对肺部恶性肿瘤的定性诊断

356例定性诊断病例中，良性肺部疾病为63例，恶性为293例。所有356例肺部肿物的SUVmax为0.5-33.3，中位值5.2。以SUVmax＞2.5为判断恶性的界值，SUVmax＞2.5者279例，SUVmax≤2.5者77例。PET/CT定性诊断后与病理行对照研究，结果为真阳性249例，真阴性33例，假阳性30例（肉芽肿性炎14例，非特异性炎症7例，结节病2例，炎性假瘤2例，真菌感染2例，其它良性病变3例），假阴性44例（腺癌32例，鳞癌5例，其它病理类型恶性肿瘤7例），假阴性中22例肿瘤直径＜1.0 cm。PET/CT定性诊断的敏感性为85.0%，特异性为52.4%，准确性为79.2%，阳性预测值为89.2%，阴性预测值为42.9%。

### 2.2 PET/CT对肺癌M分期及在术后肿瘤复发转移中的监测

275例肺癌在传统分期检查中共发现远处转移38例（38/275, 13.8%），然而PET/CT（46/275, 16.7%）较传统检查额外发现远处转移8例。包括骨转移6例，肾上腺转移1例，肝转移并骨转移1例。肺癌手术后临床怀疑复发者行PET/CT共计89例，PET/CT检查时间与手术时间间隔为1个月-93个月，中位时间为19.0个月。传统检查共发现转移及复发37例（37/89, 41.6%），PET/CT较传统检查（43/89, 48.3%）额外发现复发6例（残端复发1例，胸壁转移1例，胸膜转移1例，多发转移3例）。

### 2.3 PET/CT对原发性肺癌N分期的诊断

168例原发性肺癌行根治手术，清扫淋巴结共计610组。其中术后病理证实转移淋巴结102组，非转移淋巴结508组。术前PET/CT对淋巴结的定性诊断与术中清扫的淋巴结在解剖位置上一一对应，比对术后淋巴结病理报告，PET/CT诊断淋巴结转移真阳性37组，假阳性31组，假阴性65组，真阴性477组。PET/CT对淋巴结分期诊断的敏感性为36.3%，特异性为93.9%，准确性为84.3%，阳性预测值为54.4%，阴性预测值为88.0%（表 2）。

**2:** PET/CT诊断淋巴结转移结果 PET/CT for LN metastasis detection

PET/CT	Pathological type		Total
	Positive	Negative	
Positive	37	31	68
Negative	65	477	542
Total	102	508	610

### 2.4 肺癌原发肿瘤SUVmax与肿瘤直径间的关系

168例行术前PET/CT检查的原发性肺癌患者的肿瘤长径与短径平均值0.1 cm-7.5 cm，中位值为2.0 cm。肿瘤SUVmax为0.5-17.1，中位值为5.2。将每个肿瘤直径与其SUVmax一一对应，行相关性分析，发现肿瘤SUVmax与肿瘤直径明显相关（*P*＜0.05）。

### 2.5 PET/CT对肿瘤化疗的疗效评价

10例化疗前后行PET/CT检查的患者，其局部肿瘤化疗前后的SUVmax见表 3，化疗前SUVmax值为1.4-13.9，平均值为7.7±3.8；化疗后SUVmax值为0.9-8.4，平均值为4.2±2.6。化疗前后SUVmax之间差异有统计学意义（*P*＜0.05）。化疗前后SUVmax下降平均百分比为（37.5±39.3）%。以化疗前后SUVmax下降50%为界值，化疗有效者5例，平均SUVmax下降百分比为（63.7±12.5）%；无效者5例，平均SUVmax下降百分比为（11.3±40.1）%。按照RECIST（Response Evaluation Criteria in Solid Tumors）标准评效，达到部分缓解（paitial response, PR）患者5例，平均SUVmax下降百分比为（56.1±20.7）%，疾病稳定（stable disease, SD）的患者5例，平均SUVmax下降百分比为（18.8±46.8）%。临床评效PR的患者与评效SD的患者化疗前后SUVmax下降百分比之间的差异有统计学意义（*P*＜0.05）。

**3:** 10例患者治疗疗效评价结果 Summary on treatment evaluation of 10 patients

No	Pretreatment SUVmax	Posttreatment SUVmax	%ΔSUVmax	PET/CT evaluation	Clinical evaluation
1	7.0	8.4	-20.0	Nonresponser	SD
2	6.0	0.9	85.0	Responser	PR
3	9.4	5.6	40.4	Nonresponser	PR
4	2.8	1.5	46.4	Nonresponser	SD
5	8.3	3.6	56.6	Responser	SD
6	13.9	5.8	58.3	Responser	PR
7	1.4	2.0	-42.9	Nonresponser	SD
8	7.4	3.4	54.1	Responser	SD
9	11.4	7.7	32.5	Nonresponser	PR
10	9.8	3.5	64.3	Responser	PR
Mean	7.7	4.2	37.5	-	-
SD: stable disease; PR: partial response.					

## 3 讨论

目前，PET/CT在肺癌的诊治中的应用日渐广泛，无论是在定性、分期、疗效评估还是术后随访中都起到了重要的作用。虽然PET/CT将解剖和功能相结合，但由于分辨率、部分容积效应及良性疾病等干扰因素，也存在一定的假阳性和假阴性^[1]^。其临床应用价值的某些方面尚有争议^[2]^。本研究总结了419例患者PET/CT检查结果，与临床、病理及随访进行对照研究，分析PET/CT在肺部肿瘤及肿瘤样病变中的应用价值。

### 3.1 PET/CT能够有效鉴别肺部良/恶性病变

除外科术后病理诊断外，临床常用的判断肺部肿瘤或肿瘤样病变良恶性的方法有超声、CT、气管镜、CT引导穿刺活检等。CT应用最为广泛，但特异性较低，假阳性率高。气管镜、CT引导穿刺等属有创检查，亦存在假阴性及假阳性。PET/CT是一种无创检查方法，适用范围广，准确性较高^[3]^。如Allen等^[4]^认为PET/CT在肺部结节定性诊断方面优于CT。Hashimoto等^[5]^的研究以SUVmax＞2.5作为诊断恶性病变的标准，认为PET/CT定性诊断的敏感性和特异性分别为100%和63%。本组PET/CT定性诊断（SUVmax＞2.5）的敏感性为85.0%，特异性为52.4%，准确性为79.2%，阳性预测值为89.2%，阴性预测值为42.9%。假阳性30例，假阴性44例。原因可能为PET/CT在感染和炎症病变亦存在高摄取可能导致假阳性结果^[4]^，建议此类患者常规抗生素治疗后再行PET/CT检查。腺癌患者肿瘤摄取程度较低^[6]^，亦受肿瘤大小影响（假阴性中22例肿瘤直径＜1.0 cm），因＜1.0 cm肿瘤受部分容积效应的影响摄取率较低，可能造成假阴性结果^[1]^。

### 3.2 PET/CT是M分期及术后转移复发监测的有效手段

导致肺癌生存率较低的重要原因之一是转移与复发。术前发现转移灶可避免不必要的手术。PET/CT可提供准确的全身信息，早期发现结构学无改变的仅存在功能学改变的隐匿转移灶，为患者的术前分期提供更多的辅助信息，改变原有的分期^[3]^。Kanzakia等^[7]^对241例术后患者进行PET/CT检查，发现PET/CT可准确的诊断肺癌复发，其敏感性、特异性、准确性、阳性预测值、阴性预测值分别为97%、96%、96%、81%和99%。Ibeas等^[3]^的研究中PET/CT较传统检查额外发现了11%的远处转移。本组研究中PET/CT较传统检查在术前额外发现了8例远处转移肿瘤，使分期提升。PET/CT在术后复查的患者中亦额外发现6例复发，使患者获得了早期治疗。我们认为PET/CT在诊断远处转移方面明显优于传统检查。

### 3.3 PET/CT在肺癌N分期中的价值

肺癌的N分期一直是胸外科备受关注的问题，常用手段为CT，其以淋巴结直径＞1.0 cm作为判定淋巴结转移的标准^[8]^，但淋巴结直径与术后病理是否转移并不一致。单一形态学信息区分转移与非转移淋巴结存在很大局限性。PET/CT将结构与功能相结合，可为肿瘤的N分期提供有价值的参考信息。然而，多数作者认为PET/CT在肺癌N分期方面的敏感性较低。Darling等^[9]^的研究认为PET/CT在纵隔淋巴结分期方面的特异性和阴性预测值较高，但有一定的假阳性率。Lv等^[10]^也得出了相同的结论。本组研究中PET/CT在肺癌N分期方面的特异性（93.9%）和阴性预测值（88.0%）较高，但敏感性较低（36.3%）。我们认为PET/CT能够有效排除非转移性淋巴结。但是，PET/CT诊断肺癌淋巴结转移的假阳性率较高，可能原因为PET/CT在肉芽肿性炎、非特异性炎症等情况下也可表现为高摄取^[9]^。

### 3.4 PET/CT在肿瘤直径方面有助于肺癌的T分期

肺癌大小及与周围组织的关系决定着肺癌的分期及疗效。PET/CT能精确地区分肿瘤组织与周围组织的界限，特别是存在肺不张与周围组织炎症时，有助于肿瘤的精确T分期，并可在肿瘤放疗靶区的划定方面发挥重要的作用^[11]^。如Pawaroo等^[11]^认为PET在周围有阻塞和不张时比CT更能有效区分T1期和T2期肿瘤。本研究的结果发现肿瘤SUVmax与肿瘤直径明显相关（*P*＜0.05），可为肺癌的T分期提供有用的辅助信息。

### 3.5 PET/CT可用作肺癌化疗评效的有效手段

肺癌化疗评效常用手段为CT，采用RECIST标准，能够有效判断肿瘤大小的变化。但CT对肿瘤化疗后大小无变化或变化甚微，而仅功能学改变的患者中应用价值有限。PET/CT能够早期预测肿瘤在结构学发生变化之前发生的功能学变化，有助于化疗的评效。Huang等^[12]^的研究发现放化疗前后PET/CT可有效评估进展期肺癌放化疗的疗效。Christoph等^[13]^报道发现病理退缩2b级（＜10%残余肿瘤细胞）和3级（无肿瘤残余）SUVmax下降明显大于2a级（＞10%残余肿瘤）。Lee等^[14]^的研究发现化疗1周期后PET/CT较传统检查可更早期预测疾病进展，避免无效的化疗。本研究结果与文献报道一致，PET/CT能够有效地区分化疗有效者与无效者，可为患者进一步治疗方案的选择提供有价值的辅助信息。

综上所述，PET/CT作为一种新型无创的检查方式可有效地鉴别肺部肿瘤及肿瘤样病变的良恶性。作为肺癌TNM分期的手段之一，PET/CT在肺癌M分期中的作用优于传统检查，也可作为术后复查的常规手段之一。PET/CT对淋巴结转移的诊断特异性较好，但敏感性不高。PET/CT在肺癌化疗评效中有积极意义。

## References

[1] Padma S, Sundaram PS, George S (2011). Role of positron emission tomography computed tomography in carcinoma lung evaluation. J Cancer Res Ther.

[2] Thomsona D, Hulseb P, Loriganc P (2011). The role of positron emission tomography in management of small cell lung cancer. Lung Cancer.

[3] Ibeas P, Cantos B, Gasent JM (2011). PET-CT in the staging and treatment of non-small-cell lung cancer. Clin Transl Oncol.

[4] Allen TL, Kendi AT, Mitiek MO (2011). Combined contrast-enhanced computed tomography and 18-fluoro-2-deoxy-D-glucose-positron emission tomography in the diagnosis and staging of non-small cell lung cancer. Semin Thorac Cardiovasc Surg.

[5] Hashimoto Y, Tsujikawa T, Kondo C (2006). Accuracy of PET for diagnosis of solid pulmonary lesions with ^18^F-FDG uptake below the standardized uptake value of 2.5. J Nucl Med.

[6] Chiu CH, Yeh YC, Lin KH (2011). Histological subtypes of lung adenocarcinoma have differential ^18^F-Fluorodeoxyglucose uptakes on the positron emission tomography/computed tomography scan. J Thorac Oncol.

[7] Kanzakia R, Higashiyama M, Maeda J (2010). Clinical value of F18-fluorodeoxyglucose positron emission tomography-computed tomography in patients with non-small cell lung cancer after potentially curative surgery: experience with 241 patients. Interact Cardiovasc Thorac Surg.

[8] Fischer BM, Mortensen J, Hansen H (2011). Multimodality approach to mediastinal staging in non-small cell lung cancer. Faults and benefits of PET-CT: a randomised trial. Thorax.

[9] Darling GE, Maziak DE, Inculet RI (2011). Positron emission tomography-computed tomography compared with invasive mediastinal staging in non-small cell lung cancer. J Thorac Oncol.

[10] Lv YL, Yuan DM, Wang K (2011). Diagnostic performance of integrated positron emission tomography/computed tomography for mediastinal lymph node staging in non-small cell lung cancer. J Thorac Oncol.

[11] Pawaroo D, Cummings NM, Musonda P (2011). Non-small cell lung carcinoma: accuracy of PET/CT in determining the size of T1 and T2 primary tumors. AJR Am J Roentgenol.

[12] Huang W, Zhou T, Ma L (2011). Standard uptake value and metabolic tumor volume of ^18^F-FDG PET/CT predict short-term outcome early in the course of chemoradiotherapy in advanced non-small cell lung cancer. Eur J Nucl Med Mol Imaging.

[13] Christoph P, Sabine L, Dirk T (2006). Value of ^18^F-Fluoro-2-Deoxy-D-Glucose-positron emission tomography/computed tomography in non-small-cell lung cancer for prediction of pathologic response and times to relapse after neoadjuvant chemoradiotherapy. Clin Cancer Res.

[14] Lee HD, Kim SK, Lee HY (2009). Early prediction of response to first-line therapy using integrated ^18^F-FDG PET/CT for patients with advanced/metastatic non-small cell lung cancer. J Thorac Oncol.

